# Recurrent Ameloblastic Fibroma of the Posterior Maxilla With Radiopaque Foci: A Diagnostic Challenge Within the Odontogenic Tumor Spectrum

**DOI:** 10.7759/cureus.108780

**Published:** 2026-05-13

**Authors:** Maria Sabbagh, Claude Chaanine

**Affiliations:** 1 Oral Surgery, Saint Joseph University of Beirut, Beirut, LBN

**Keywords:** ameloblastic fibroma, diagnostic challenge, odontogenic tumor, radiopaque structure, recurrent

## Abstract

Ameloblastic fibroma (AF) is classically defined as a benign mixed odontogenic tumor lacking calcified dental tissues and typically presenting as a radiolucent lesion in the posterior mandible of young patients. This case report presents a recurrent AF of the posterior maxilla in a 16‑year‑old girl, characterized by radiopaque foci and a discordance between radiological and histopathological findings. A panoramic radiograph and cone-beam computed tomography (CBCT) initially suggested an odontoma indirectly interfering with the eruption of tooth 27. Surgical enucleation revealed a radiolucent lesion containing distinct radiopaque structures and multiple gelatinous fragments, all removed and submitted for histopathological analysis. Repeated histopathological evaluations twice confirmed the diagnosis of AF without evidence of organized dental hard tissue or malignancy. Five years later, a routine follow‑up CBCT revealed a similar lesion at the same site, again managed by surgical enucleation and again diagnosed as AF. This case challenges current concepts in the World Health Organization odontogenic tumor classification, particularly the separation of AF from developing odontomas, and underscores the diagnostic complexity created by internal calcifications in lesions histologically lacking dental hard tissue. The findings emphasize the critical importance of extensive histopathological sampling and close clinico‑radiologic-pathologic correlation in atypical or recurrent mixed odontogenic tumors. It is also suggested that AF may occasionally exhibit limited inductive or calcific features, without justifying reclassification within the developing odontoma spectrum.

## Introduction

According to the World Health Organization (WHO) Classification of Head and Neck Tumors, ameloblastic fibroma (AF) is an uncommon, benign, mixed epithelial and mesenchymal odontogenic tumor [1]. It presents in both epithelial and mesenchymal odontogenic tissue and lacks the presence of any calcified dental structure [2]. It commonly arises in the mandible during the first two decades of life and shows a slight male predilection. It is frequently found in association with an unerupted or impacted tooth [3]. Radiologically, the lesion appears as a unilocular or multilocular radiolucent image with sclerotic borders, where smaller lesions tend to be unilocular [4]. Macroscopically, the lesion presents as a solid soft-tissue mass with a smooth surface. Histologically, AF shows a true biphasic pattern of neoplastic odontogenic epithelium and ectomesenchyme. The epithelium forms strands and islands within a cellular, myxoid stroma resembling primitive dental papilla, with plump stellate and ovoid fibroblasts in a loose connective matrix similar to developing dental pulp [5]. According to the literature, if the lesion presents histologically dentinoid material, it should be classified as ameloblastic fibrodentinoma (AFD), and if any enamel matrix is found, it should be classified as ameloblastic fibro-odontoma (AFO) [6]. Nevertheless, AFO and AFD have been excluded from the WHO classification since 2017, as they were found to be developing odontomas, even though the presence of the *BRAF p.V600E* mutations makes them similar to AF and different from odontoma, which may suggest the neoplastic nature of these lesions [1].

This case report presents a histologically confirmed AF in a 16-year-old girl who presented with atypical hard tissue formation that appeared on radiographs as a radiopaque structure.

## Case presentation

A 16-year-old girl was referred by her orthodontist to have a surgical procedure to remove what seemed to appear on the panoramic radiograph as an odontoma, blocking the evolution of tooth 27. A cone-beam computed tomography (CBCT) scan was performed the same day to evaluate the surgical approach. The scan showed a unilocular radiolucent image distally to tooth 26, along with radiopaque structures under the occlusal surface of tooth 27, that might be indirectly interfering with tooth eruption (Figure 1).

**Figure 1:**
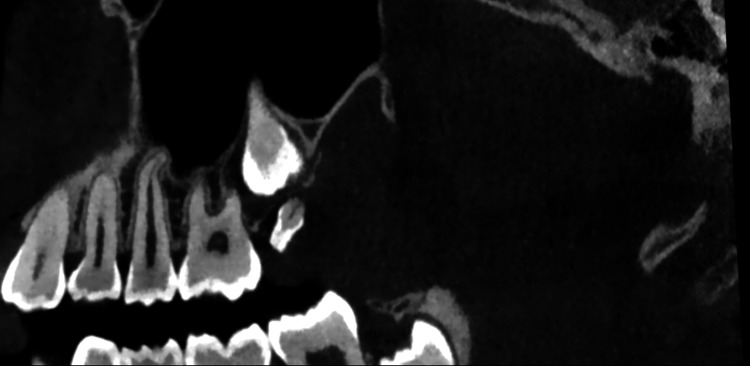
Panoramic view of a cone-beam computed tomography scan showing the radiolucent image that includes a radiopaque structure indirectly interfering with the evolution of tooth 27.

Surgical enucleation of the posterior radiolucent lesion was performed under appropriate local anesthesia. The lesion extended posteriorly toward the pterygoid region and superiorly to the maxillary sinus. Intraoperatively, two radiopaque structures were identified and removed. The lesion was associated with several gelatinous masses that were clearly separated from one another and varied in size and consistency. These masses were friable and proved difficult to grasp with toothed forceps (Figure 2).

**Figure 2:**
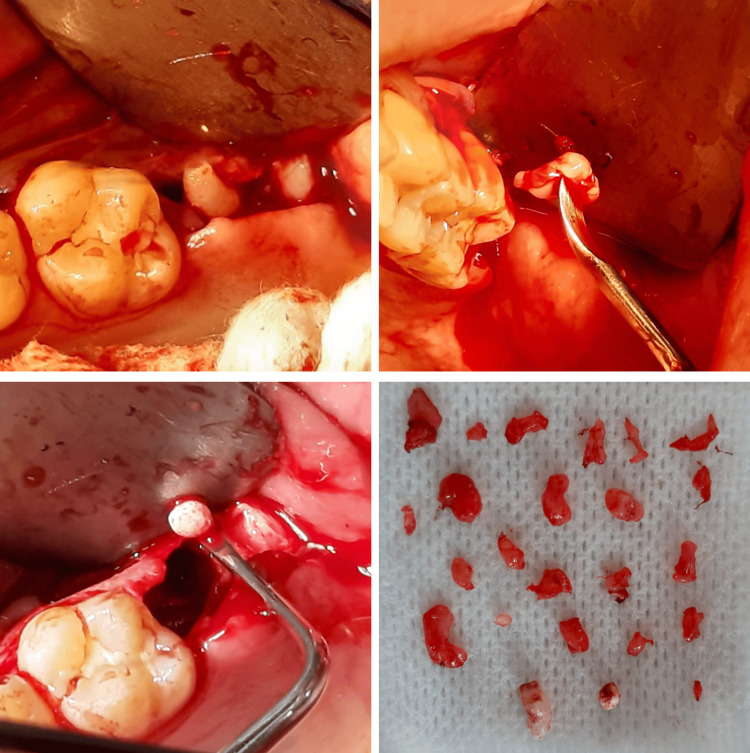
Intraoral photos showing multiple fragments of different consistencies.

After complete removal of the lesion, the surgical cavity was carefully inspected and curetted. The Valsalva maneuver was performed and was negative, with no evidence of oroantral communication.

The mucoperiosteal flap was repositioned and sutured with 4-0 silk. Postoperative management included antibiotic therapy with amoxicillin-clavulanic acid and a nonsteroidal anti-inflammatory drug (ibuprofen). The excised specimen was submitted for histopathological examination. The histopathological examination confirmed AF with no signs of malignancy (Figure 3).

**Figure 3:**
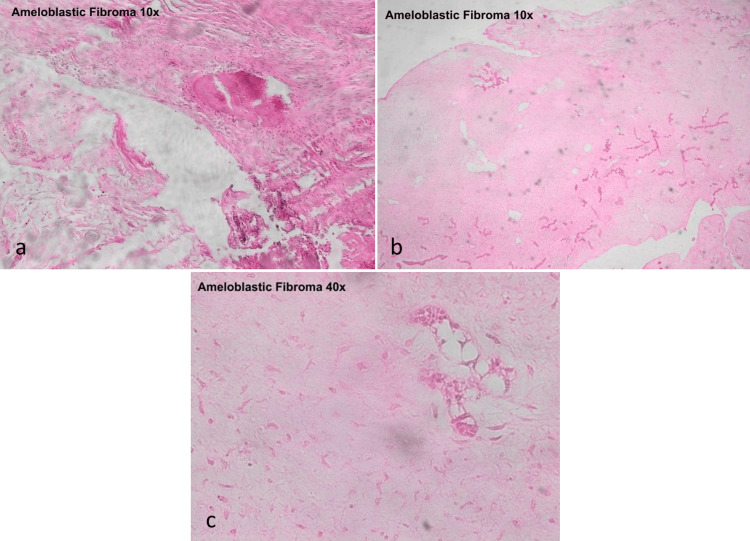
Histological sections of ameloblastic fibroma showing three representative slices (a-c) stained with hematoxylin and eosin at original magnifications ×10, ×10, and ×40, respectively.

Given the inconsistency between the histopathological findings and the clinical presentation, a second histopathological evaluation was requested. The repeat examination confirmed the initial diagnosis of AF. A follow-up CBCT scan was ordered after three months (Figure 4) and showed the start of the evolution of tooth 27 occlusally. Another follow-up was performed one year later (Figure 5), which showed tooth 27 migrating more occlusally.

**Figure 4:**
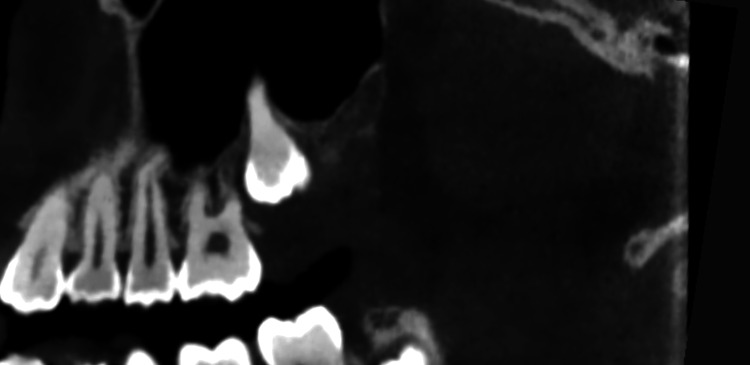
Panoramic view of a cone-beam computed tomography scan showing the three-month follow-up scan.

**Figure 5:**
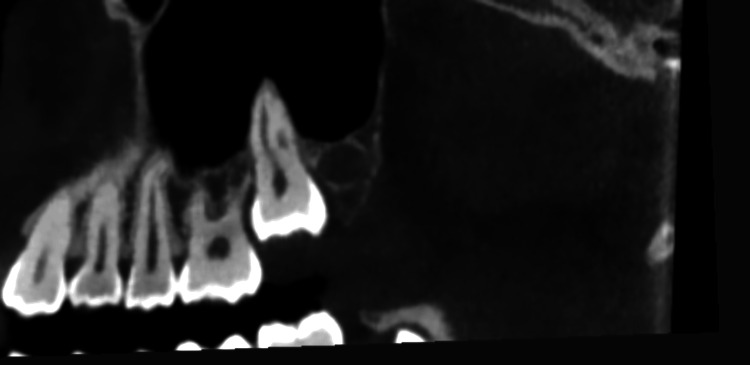
Panoramic view of a cone-beam computed tomography scan showing the one-year follow-up scan.

After the eruption of the tooth, the patient did not come back for a follow-up. Five years later, she presented to the clinic to extract her wisdom teeth, and during the routine CBCT scan, a radiopaque structure with a radiolucent image surrounding it was identified at the location of the previous AF lesion (Figure 6).

**Figure 6:**
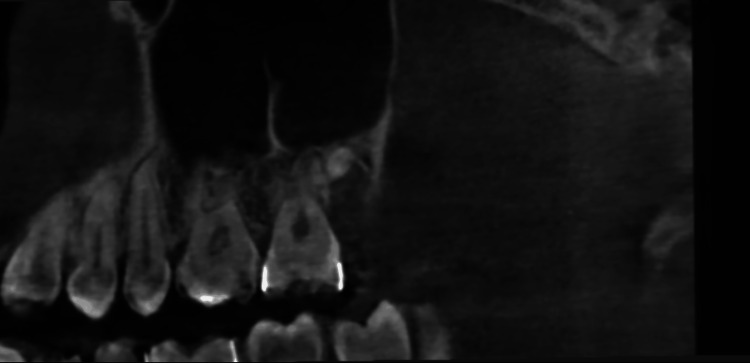
Panoramic view of a cone-beam computed tomography scan showing the five-year follow-up scan.

Surgical enucleation of the lesion was performed to remove the structures that appeared identical to the previous ones five years earlier. The histopathological examination also came back as an AF. One year later, the patient refused to come back for a follow-up, claiming she was feeling well.

## Discussion

An AF, as previously mentioned, is a mixed odontogenic benign tumor, presenting both epithelial and mesenchymal tissue, and lacks the presence of any dental hard tissue [2]. It usually develops during the first two decades of life in the posterior mandibula and appears as a radiolucent image without any radiopaque components [3].

However, the case presented differs from the description of AF in the literature in many ways. It presented in the posterior maxilla rather than the mandible, and presented hard dental tissue formation, regardless of the multiple histopathological confirmations of the lesion being a true AF.

In the literature, it has been suggested that if dentinoid or enamel matrix is found, the lesion would be classified as AFD or AFO, respectively [6]. However, since the 2017 WHO classification, both AFD and AFO are no longer recognized as separate entities, but rather classified as developing odontomas [7]. This modification remained the same in the 2022 WHO classification, with AF still classified as a mixed benign odontogenic tumor [1].

Chen et al. suggested that AF, AFD, AFO, and odontomas are merely different stages of the same lesion. Although this suggestion was disputed in the literature, some studies have supported the possibility of a true AF neoplasm, especially in younger patients, to be a primitive stage of a developing odontoma [8].

While AF may present inductive properties, it does not always result in mature dental tissue formation [2]. Ver Berne et al. emphasized the importance of the organization of the hard tissue [9]. They suggested that if the lesion lacks mature dental hard tissue and has irregular mineralization, it should remain a neoplastic AF, while the presence of mature enamel and tubular dentin reflects the hamartomatous non-neoplastic behavior of an odontoma [9].

In the present case, the recurrence of the lesion suggests the neoplastic nature of the lesion, thus aligning with the diagnosis of AF. Except that the hard dental tissue formation present in the radiograph showed distinguished dental structures, rather than irregular mineralization.

While AFD and AFO have been classified as developing odontomas, making them hamartomatous, the presence of the *BRAF p.V600E* mutation in these lesions makes them more similar to AF, suggesting the neoplastic nature of these lesions [1]. In addition, Roza et al. found that the presence of SOX9 in AF, AFD, and AFO has a potential role in odontogenic differentiation [10]. This opens the question of reclassification of ADF/AFO lesions.

An important issue that needs to be discussed is the importance of proper histopathological sampling [11]. Mixed odontogenic tumors are not uniform; different parts of the same lesion can look very different on histological sections. Variations in cell density, tissue development, and sometimes small areas of calcification can be observed. In this case, not finding dental hard tissue does not necessarily mean their absence, but rather their presence in areas that were not sampled during the histopathological analysis, especially if the radiological and clinical findings suggest the presence of calcified dental structures.

Aside from sampling issues, there are other possible explanations to consider. For example, the lesion may show only weak or incomplete inductive activity, leading to very small amounts of dentin-like material, or the calcifications seen could simply be dystrophic and not related to true tooth formation, or a separate odontoma grafted on the AF.

## Conclusions

This case highlights an unusual presentation of AF, combining recurrence with radiopaque features that created a discrepancy between radiology and histopathology. Despite this, repeated examinations confirmed the absence of organized dental hard tissue, supporting the diagnosis of AF. The internal calcifications may reflect limited inductive activity, dystrophic mineralization, or simply sampling limitations within a heterogeneous lesion. These findings underline how important it is to examine enough tissue samples and to carefully correlate clinical, radiological, and histological features, especially in atypical or recurrent cases. Overall, this case suggests that AF can occasionally show subtle inductive features without warranting reclassification within the developing odontoma spectrum, while emphasizing the need for adequate sampling and long-term follow-up.
